# Classifying Storage Temperature for Mandarin (*Citrus reticulata* L.) Using Bioimpedance and Diameter Measurements with Machine Learning

**DOI:** 10.3390/s25082627

**Published:** 2025-04-21

**Authors:** Daesik Son, Siun Lee, Sehyeon Jeon, Jae Joon Kim, Soo Chung

**Affiliations:** 1 Department of Biosystems Engineering, Seoul National University, Seoul 08826, Republic of Korea; ftiu7957@snu.ac.kr (D.S.); dltldns852@snu.ac.kr (S.L.); shjeon0304@snu.ac.kr (S.J.); 2 Research Institute of Agriculture and Life Sciences, Seoul National University, Seoul 08826, Republic of Korea; 3 Flexible Electronics Research Section, Hyper-Reality Metaverse Research Laboratory, Electronics and Telecommunications Research Institute (ETRI), Daejeon 34129, Republic of Korea; skin@etri.re.kr; 4 Integrated Major in Global Smart Farm, Seoul National University, Seoul 08826, Republic of Korea

**Keywords:** bioimpedance change, integration with diameter, storage temperature classification, machine learning, mandarin

## Abstract

Mandarin (*Citrus reticulata* L.) is consumed worldwide. Improper storage temperatures cause flavor loss and shorten shelf lives, reducing marketability. Mandarins’ quality is difficult to assess visually, as they show no apparent changes during storage. Therefore, a simple, non-destructive method is needed to assess their freshness as affected by temperature. This work utilized non-invasive bioimpedance spectroscopy (BIS) on mandarins stored at different temperatures. Eight machine learning (ML) models were trained with bioimpedance data to classify storage temperature. Also, we confirmed whether integrating diameter and time-series changes into the bioimpedance could improve the ML models’ accuracies by minimizing sample variations. Additionally, we evaluated the effectiveness of equivalent circuit (EC) parameters derived from bioimpedance data for ML training. Although slightly less accurate than using raw bioimpedance data, EC parameters can efficiently reduce data dimensionality. Among all models, the SVM model trained with changes in bioimpedance integrated with diameter data achieved the highest accuracy of 0.92. It was a significant improvement compared to the accuracy of 0.76 achieved when using only the raw bioimpedance data. Thus, this study suggests a novel method of integrating diameter and bioimpedance changes to assess the storage temperature of mandarins. This approach can also be applied to other fruits when utilizing BIS.

## 1. Introduction

Mandarin is the second most widely produced citrus variety in the world after orange [1]. Unlike other citrus fruits, such as oranges and grapefruits, which are difficult to peel, mandarins are seedless and easy to peel, which has led to a continuous increase in consumption [2]. According to a USDA investigation, 36.8 million tons of mandarins were produced in 2023, exhibiting a 13% increase compared to 2019 [3]. Mandarins have a short shelf life of 1–2 weeks at room temperature, and improper storage conditions can lead to the rapid deterioration of fruit quality, resulting in crucial economic loss [4]. Total soluble solids (TSS), total acid (TA), vitamin C, and aroma volatiles are the key components affecting the taste of mandarins [5], and these components change significantly after postharvest. While storage at higher temperatures causes flavor loss in mandarins [6], their flavor is well maintained at low temperatures (4 °C) [7], since storing mandarins at low temperatures reduces respiration, metabolic rate, and water loss, which prevents decay and overripening [8]. Therefore, maintaining an appropriate temperature during the distribution process is crucial for preserving the quality of citrus fruits [8]. Although most mandarins are stored at low temperatures of 5–8 °C immediately after packaging, they are often exposed to high temperatures during transportation to distribution centers or points of sale [6]. Because of inappropriate storage conditions, over 25% of fruits are wasted, and 20–40% of agricultural products are damaged before they reach consumers [9].

Mandarins, which turn green to orange during ripening [10], are typically distributed when they are orange. Unlike fruits such as bananas, which exhibit noticeable visual changes during the distribution process [11], it can be difficult to notice apparent visual changes in mandarins even when they are stored at high temperatures. Despite the lack of visible differences, mandarins that are exposed to high temperatures experience quality degradation and reduced shelf life, diminishing their marketability. Therefore, there is the need for a simple method to identify whether mandarins have been stored and distributed at appropriate temperatures in order to assess their value accurately. This method should be non-destructive to preserve their marketability. Several non-destructive techniques, including optical spectroscopy [12], such as Raman [13]; imaging techniques [14]; nuclear magnetic techniques [15]; and electronic noses [16], have been used to assess fruit quality. Particularly, portable Raman spectroscopy has proven effective in non-destructive assessments of mandarin freshness by analyzing carotenoid changes on the fruit’s surface [13]. Although Raman spectroscopy offers high sensitivity and is effective for on-site applications using portable devices [17,18], it can be affected by interference from ambient light, leading to noise in the data and the overlapping of Raman bands from various biomolecules complicates data analysis [19]. Furthermore, many non-destructive techniques, including Raman, primarily reflect information from a shallow surface, making its application to fruits like mandarins limited. Also, these methods often involve large or expensive equipment, which limits their application in the field.

Bioelectrical impedance spectroscopy (BIS) can be a non-invasive, rapid technology for detecting fruit quality [20,21] regardless of the ambient light. It can also be a low-cost and field-friendly method using a portable system with an impedance analyzer chip and a commercial microcontroller such as a Raspberry Pi [22]. It applies a wide frequency range of alternating current (AC) to biological tissues to obtain their impedance spectra. Electrolytes, such as the intracellular and extracellular fluids in plant cells, act as electrical resistance, and the lipid bilayer of the cell membrane is polarized in response to the applied current, acting as a capacitor [23]. This plant cell structure leads to changes in the impedance spectrum across frequencies [24]. Many studies have applied BIS to non-invasively classify the ripeness of avocados [25], tomatoes [26], strawberries [27,28], and bananas [29]. BIS has also been widely applied to diagnose freeze damage in citrus fruits, such as grapefruit [30], lemon [31], tangerine [20], and orange [32].

BIS can be represented as an electrical equivalent circuit (EC). By fitting BIS data to an EC modeled by the biological components of plant tissues, such as cell membranes and cell walls, the high-dimensional BIS data can be simplified with a few circuit elements that enable the interpretation of physiological changes [24]. While many previous studies interpret EC parameters concerning changes in fruit quality [33,34], they have not used these parameters as training inputs for fruit quality classification models. Since BIS data captures impedances and phases across wide frequency ranges, they are typically high-dimensional. Therefore, dimensionality reduction with EC parameters can be an intriguing strategy for developing effective classification models [24].

BIS is influenced by the contact resistance between the electrodes and the surface, as well as the heterogeneity of the biological tissue. Previous studies have reported variations in measurements based on location and sample [35,36]. These variations significantly degrade the data quality which leads to poor performance of machine learning (ML) models [37,38]. Therefore, to accurately assess fruit quality using BIS, focusing on the changes in bioimpedance measured from the same location over time could be beneficial. Through this, we can acquire uniform data and utilize the changes in bioimpedance to minimize the impact of variations between samples. Because fruits undergo various postharvest and distribution processes, leading to variations in their initial state, focusing on changes in bioimpedance could be a more accurate method for predicting the storage temperature of fruits.

Furthermore, given that BIS measures the complex impedance of biological tissues, the output is significantly influenced by the distance between electrodes [39]. Fruits are harvested and distributed in various sizes. Therefore, it is essential to consider the data variations due to fruit size when applying BIS to assess fruit quality. Previous studies that classified fruit ripeness or diagnosed freeze damage did not consider the variations in BIS caused by differences in fruit size. Recently, it has been proven that integrating fruit size data with BIS can improve the accuracy of classifying the ripeness of strawberries [28]. However, this study only selected a few frequencies from a wide frequency range of BIS data with the lowest correlation with fruit size, which has limited information to reflect the interaction between size and BIS data. To better reflect the impact of fruit size on BIS data, it might be appropriate to use the entire frequency range of BIS data along with fruit size for model training.

In this study, we classified the storage temperature of mandarins, which significantly affects their freshness, using ML models trained with non-invasive BIS data. We explored a novel approach by utilizing mandarin size and bioimpedance changes as features, as these factors can influence the consistency of bioimpedance data. The freshness of mandarins, which is highly affected by storage temperatures, is not easily discernible through visual inspection. BIS can assess it non-invasively in a simple method. It can help consumers select products of appropriate value during the distribution and consumption stages and assist in determining suitable pricing based on the condition of the mandarins.

## 2. Materials and Methods

### 2.1. Sample Preparation and Storage Temperature

Small and large mandarins (grown in Jeju, Korea) were purchased in equal proportions from local markets. The sizes of mandarins were distinguished by the vendor. The mandarins were acquired in two separate batches, with a 4-week interval between purchases, from different markets. Each batch accounts for 50% of the total sample set. The mandarins were fresh and ripe enough with a fully orange color to eat at the time of the purchase. Excluding bruised or damaged samples, a total of 184 mandarin samples, each comprising equal halves of large and small sizes (92 samples), were prepared. Half of each size group (46 samples) was stored at room temperature (21 °C), and the other half (46 samples) was stored in a refrigerator at 4 °C (both conditions were in low relative humidity conditions from 25 to 40%). Therefore, all 184 samples are classified into four groups, each containing 46 samples. All samples were stored for 48 h, placed in meshed baskets to ensure ventilation with the air, and were not stacked to avoid physical damage due to their weight.

### 2.2. Data Acquisition

#### 2.2.1. Bioimpedance Measurements

**Figure 1:**
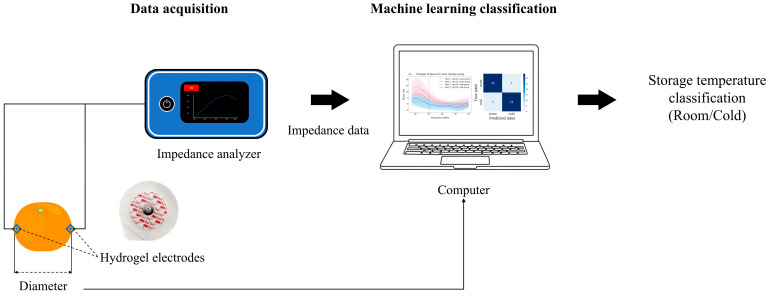
Experimental setup for acquiring bioimpedance data from a mandarin sample using an impedance analyzer and a pair of hydrogel electrodes. BIS was conducted in a direction perpendicular to the stem of the mandarin, and the diameter data were collected separately.

Bioimpedance was measured on the surface of each mandarin using an impedance analyzer (PalmSens4, PalmSens, Houten, The Netherlands) and two electrocardiogram hydrogel electrodes (2223H, 3M, Saint Paul, MN, USA) as shown in Figure 1. Bioimpedance data were obtained from the maximum distance for each sample by attaching a pair of hydrogel electrodes to both ends of each mandarin surface in the direction of the largest diameter.

BIS was conducted across the frequency range from 50 Hz to 1 MHz, covering 30 data points on a logarithmic scale. An AC power supply of 500 mV was applied between two electrodes using the PSTrace 5.9 software (PalmSens, Houten, The Netherlands). Bioimpedance includes impedance and phase angle at each of the 30 frequencies. The impedance and phase angle can be described by Equations (1) and (2). Z and θ represent impedance and phase angle, respectively. The impedance consists of a real part ($Z_{real}$) and an imaginary part ($Z_{img}$).

(1) $$Z=Z_{real}+jZ_{img}$$

(2) $$\theta =arctan\left(\frac{Z_{img}}{Z_{real}}\right)\frac{180^{\circ }}{\pi }$$

BIS was conducted every 24-h interval over 48 h (day 0, day 1, and day 2). Therefore, 552 bioimpedance data (46 samples × 3 days × 2 sizes × 2 temperatures) were collected. Here, 184 bioimpedance of the initial state (day 0) was only used to calculate changes in bioimpedance by day. For the cold group, the samples were left at room temperature for 3 h just before the measurement to measure bioimpedance at a temperature similar to that of the room group. After being left at room temperature for 3 h, the core temperature of the cold group was the same as that of the room group. All samples were labeled to obtain continuous data for each mandarin for three measurements (day 0, day 1, and day 2), and the measurement locations for bioimpedance were marked to acquire data at a consistent location. Before each measurement, the mandarins were wiped to remove any moisture on the surface.

#### 2.2.2. Diameter Measurements

The diameter of all mandarin samples was measured with a Vernier caliper at the point where the diameter was largest, with the stem as the center. The measured diameters were used in the subsequent ML classification model to improve the classification accuracy with the changes in bioimpedance.

#### 2.2.3. Equivalent Circuit Fitting

**Figure 2:**
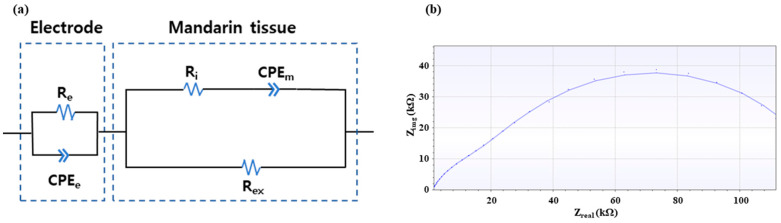
(**a**) Equivalent circuit of mandarin tissue and electrode parts to fit the bioimpedance data, (**b**) An example of fitted results with lowest accuracy ($\chi^{2}$ = 3 × 10^−4^) representing the actual data (dot) and fitted result (solid line).

The acquired bioimpedance data were fitted to an EC, shown in Figure 2, using the PSTrace 5.9 software. The EC includes the electrode part, which represents the contact resistance between the electrodes and the surface of the mandarin, and the internal tissue part, consisting of extracellular and intracellular fluids, as well as the cell membrane. We added the electrode part in series with the mandarin tissue part (modified Hayden model) as suggested by previous research [40]. The constant phase element (CPE) represents the non-ideal capacitive behavior of contact resistance (CPE_e_) and cell membrane (CPE_m_), composed of non-ideal capacitance (Q) and ideality factor (α) as shown in Equation (3). Since the plant cell membrane, unlike electrical elements, cannot represent a stable capacitor, capacitors in the EC of plant tissue have been replaced by a CPE in recent studies [24]. In this context, the ideality factor ranges between 0 and 1, with a value of 1 indicating an ideal capacitor. R represents the contact resistance (R_e_), intercellular resistance (R_i_), and extracellular resistance (R_ex_). The impedance and phase of bioimpedance at 30 frequency points are fitted to the five EC elements in Figure 2a, resulting in seven EC parameters due to the CPE having two parameters. All 552 bioimpedance data were fitted to the EC accurately with chi-square ($\chi^{2}$) values below 3 × 10^−4^ calculated by PSTrace 5.9 software. As shown in Figure 2b, even in the case with the lowest fitting accuracy, there is no noticeable difference between the actual data (dots) and the fitted result (solid line). These seven EC parameters were used as input for subsequent ML models to compare with the performance of raw bioimpedance data in classification accuracy.

(3) $$Z_{CPE}=\frac{1}{Q({\omega j)}^{\alpha }}$$

### 2.3. Machine Learning Classifications

#### 2.3.1. Data Preprocessing

All bioimpedance (30 impedance and phase, respectively) and seven EC parameters for 552 data were normalized at each feature point to prevent skewness toward features with larger scales, thus ensuring uniform variance across the dataset. Also, to minimize errors by variations in the initial states of each sample, we acquired the changes in bioimpedance and EC parameters between the data measured at the initial state and the data measured at 24 h (day 1) and 48 h (day 2). The changes were calculated as shown in Equation (4). X represents the feature type, and t represents the storage time, where $X_{0}$ is the initial value of each feature. The diameter of the mandarin was also normalized and used as an input for ML along with the bioimpedance data or EC parameters.

(4) $$\Delta X_{t}=X_{t}-X_{0},\;\;\;\;\;\;t\;\in \{24,\;48\}$$

#### 2.3.2. Machine Learning Models

In this study, we employed eight ML models (Support vector machine (SVM), Linear Regression (LR), Multi-layer perceptron (MLP), k-nearest neighbors (kNN), Random Forest (RF), Linear Discriminant Analysis (LDA), Naïve Bayes (NB), Decision Tree (DT)) to compare the performance in classifying storage methods. All ML models were performed using the sci-kit learn library (https://scikit-learn.org/stable/ accessed on 22 January 2025) in Python (version 3.11.12).

#### 2.3.3. Datasets for Machine Learning Model

In order to confirm the optimum data types for ML models to classify two storage groups (room and cold), we compared the performance of eight ML models using the following six types of datasets: bioimpedance-based dataset (raw bioimpedance, changes in bioimpedance, changes in bioimpedance with diameter) and EC parameter-based dataset (raw EC parameter, changes in EC parameter, changes in EC parameter with diameter). As summarized in Table 1, the bioimpedance data were acquired at 30 frequency points for both impedance and phase, giving a total of 60 features. The EC parameter data has seven features, as described in Section 2.2.3. For changes in the bioimpedance and EC parameter datasets, they have the same number of features, and the difference from the initial value measured on day 0 by Equation (4), was used. For the dataset combined with diameter, one additional feature is included, resulting in 61 and eight features, respectively. Each dataset has 368 data instances measured from 184 mandarin samples on day 1 and day 2

#### 2.3.4. Model Tuning and Performance Evaluation for Machine Learning Classifier

We divided all 368 data for all datasets into training and test datasets in a ratio of 8:2. To select the optimal hyperparameters for each ML model, the accuracies and standard deviations of eight ML models were compared using 5 times repeated 10-fold cross-validation (CV) with the training dataset. The hyperparameters were determined using the grid search method, selecting the combination that resulted in the highest accuracy for 5 times repeated 10-fold CV. The range of hyperparameters for each ML model used in the grid search is shown in Table S1.

After selecting the hyperparameters with the highest accuracy, we compared the same ML models trained on different datasets. To assess whether the performance differences were statistically significant, we used the Wilcoxon signed-rank test, which does not assume independence between paired samples.

Among the models that showed statistically significant improvements in performance, we obtained the confusion matrices for the training and test sets using the model that achieved the highest accuracy in 5 times repeated 10-fold CV. Precision, recall, and F1-score were calculated for two confusion matrices to identify the consistency of the model’s performance. The F1-score is the harmonic mean of precision and recall. Precision is the ratio of true positives among the samples predicted as positive. At the same time, recall is the proportion of true positives that the model correctly identifies out of all actual positives. All performance evaluation metrics for the ML models were calculated as Equations (5)–(8). The symbols T, F, P, and N represent true, false, positive, and negative, respectively.

(5) $$Accuracy=\frac{TP+TN}{TP+TN+FP+FN}$$

(6) $$Precision=\frac{TP}{TP+FP}$$

(7) $$Recall=\frac{TP}{TP+FN}$$

(8) $$F1\;score=2\times \frac{Precision\times Recall}{Precision+Recall}$$

## 3. Results and Discussion

### 3.1. Bioimpedance and Size Distribution

**Figure 3:**
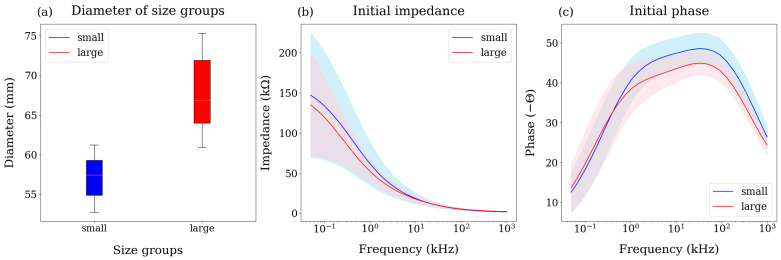
(**a**) Diameter of different size groups (blue for small, red for large). (**b**) Impedance and (**c**) phase patterns of different size groups for small (blue) and large (red) at the initial state (day 0). The standard deviations are represented as shaded areas.

The diameters of the samples were categorized into two distinct size distributions, as shown in Figure 3a. Since the bioimpedance data represent the complex resistance of the path where the current flows, they could be influenced by the size of the mandarins. In order to confirm the effect of size on the bioimpedance data, we measured the bioimpedance of all the samples in their initial state, as shown in Figure 3b,c. No distinct differences or trends related to mandarin size were observed in the magnitude of the impedance (Figure 3b). On the other hand, the phase patterns changed distinctly by mandarin size. The average phase of the small size (blue) was higher than that of the large size (red). The phase of the complex impedance approaches −90° as the imaginary part increases while the real part decreases, and approaches 0° when the opposite occurs. As fruits enlarge, the cell membrane enlarges, and the cell density decreases [41]. Since the total capacitance of the cell membrane is proportional to its surface area [42], an increase in capacitance could be associated with a decrease in reactance, the imaginary part of the impedance, and the absolute phase value by Equation (2). Therefore, when understanding the fruit based on bioimpedance spectroscopy, it would be beneficial to consider the fruit’s diameter data.

**Figure 4:**
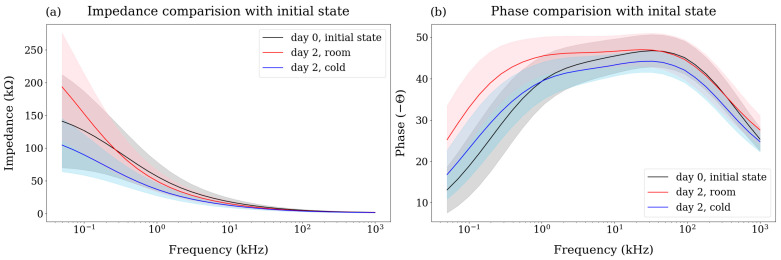
(**a**) Impedance and (**b**) phase patterns on day 2 for storage groups, namely room (red) and cold (blue), comparing initial state (black). The standard deviations are represented as shaded areas.

Mandarins stored at room temperature exhibited an increase in impedance due to water loss in Figure 4a. When plant cells lose water, the surface area of the cell membrane decreases, and its thickness may increase [43]. Since capacitance is directly proportional to the cross-sectional area of the cell membrane and inversely proportional to its thickness, the mandarins stored at room temperature exhibited a decrease in capacitance and an increase in reactance, resulting in an overall increase in the phase angle magnitude, which is more pronounced in the low-frequency range (Figure 4b). This is because the current can penetrate the cell membrane at high frequencies. These changes in impedance and phase are consistent with previous studies observing impedance variations during the ripening of mandarins [44].

We compared the pattern of the bioimpedance data at the initial (day 0) with the final (day 2) state for each storage group in Figure 4. As mentioned in materials and method, the BIS for the cold storage mandarins was conducted after leaving them at room temperature for 3 h ensuring the same core temperature for both storage groups to avoid any effects of temperature on the bioimpedance data. For the magnitude of the impedance (Figure 4a), the standard deviation of the two storage groups (red and blue) and initial state (black) overlapped significantly, resulting in insufficient separation between the groups. Although the standard deviation of the phase also overlapped to some extent in Figure 4b, the cold group and the initial state showed a similar trend being lower than the room group. While these differences in pattern suggest the potential for classifying storage conditions, the high standard deviation (shaded in color) of the obtained data makes it difficult to distinguish between groups accurately.

### 3.2. Changes in Bioimpedance

**Figure 5:**
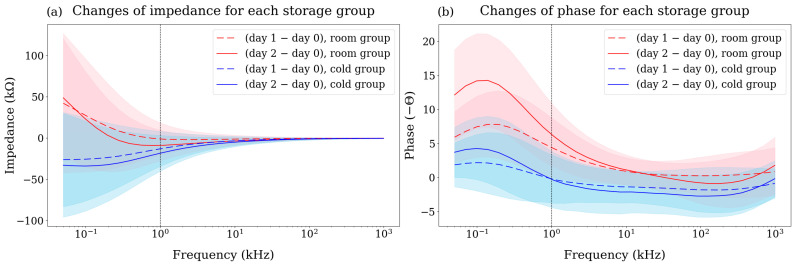
Changes of (**a**) impedance and (**b**) phase patterns of day 1 (dashed line) and day 2 (solid line) for two storage groups, namely room (red) and cold (blue). The standard deviations are represented as shaded areas.

To exclude sample variations, we observed the changes in bioimpedance over the storage period for each sample as shown in Figure 5. The initial impedance and phase values may vary for each sample, but by observing these changes, we were able to reduce this variation. For the changes in the impedance (Figure 5a), two groups were separated distinctly compared with raw bioimpedance data (Figure 4a). Also, the two groups exhibited different distributions across all frequency ranges for the phase changes (Figure 5b). While the room group showed a significant difference between day 1 and day 2, the cold group showed similar patterns and was distributed near zero. This result implies that the cold group maintained its initial state well compared to the room group.

**Figure 6:**
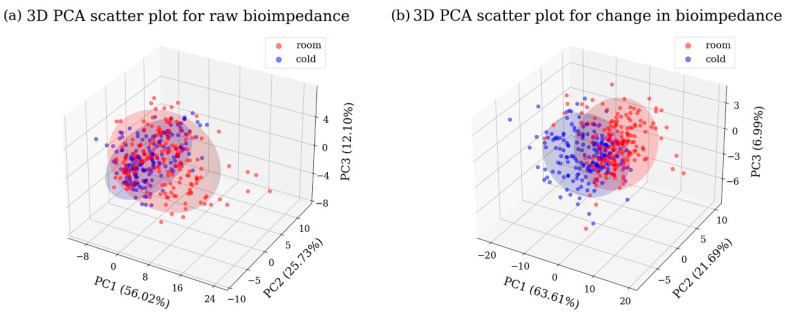
3D PCA scatter plots for (**a**) raw bioimpedance and (**b**) changes in bioimpedance. The room and cold groups are represented by red and blue dots respectively.

To visually show the data distribution between the two groups, principal component analysis (PCA) was performed, and Figure 6 shows the 3D scatter plots of the first three principal components, which explain 93.85% and 92.29% of the total variance respectively. As shown in Figure S2, statistical analysis of the features influencing PC1, which reflects the highest variance in the data, revealed that impedance below 1 kHz had the statistically significant highest loading value. Compared to the raw bioimpedance data (Figure 6a), the changes in bioimpedance (Figure 6b) showed that the two groups can be more clearly distinguished. Therefore, these visualizations suggest that the classification accuracy could be improved using the changes in bioimpedance.

### 3.3. Storage Temperature Classification with Machine Learning

In order to determine the hyperparameters of each ML model, we performed the 5 times repeated 10-fold CV using the grid search method on the training data (80% of the entire data) with the hyperparameters presented in Table S1. The hyperparameters that resulted in the highest accuracy for each model were selected. The impact of hyperparameter combinations on accuracy for each ML model is presented in Table S2 and the determined hyperparameters for each model are presented in Table S3.

To determine the best ML model and dataset for classifying two groups (room and cold), the storage temperature of mandarins, we compared the 5 times repeated 10-fold CV accuracies of eight ML models with six types of datasets in Table 2. For the raw bioimpedance-based dataset (row 1), SVM, LR, and MLP showed relatively higher accuracy with 0.81, 0.77, and 0.79 respectively.

When using the changes in bioimpedance (row 2), the accuracy was significantly improved in most ML models except for LDA, compared with raw bioimpedance. Especially, most non-linear models such as kNN, RF, and DT showed notable increases in accuracy. As they were trained with the changes in bioimpedance with respect to the initial state (day 0), the variations between samples due to the diverse initial states of mandarins would be corrected, focusing more on the changes in bioimpedance data for the different storage temperatures. Using these changes in bioimpedance, the accuracy of the SVM model increased from 0.81 to 0.85 showing the best performance. In some previous studies applying BIS to plants, the SVM model was effective [23,25], whereas in another study, MLP outperformed SVM [28]. Therefore, when using machine learning models to diagnose plants based on bioimpedance data, it is important to optimize and compare the hyperparameters of various models to select the most suitable one.

For further improvement, we incorporated the diameter data with the bioimpedance change to reflect the impact of the fruit’s size on the bioimpedance data (row 3). The accuracy of the SVM model and the MLP model showed the best performance, rising from 0.85 to 0.86 and from 0.83 to 0.86 respectively. Because the diameter, the size of mandarin, could affect changes in the conductive path and surface area of cell membranes [41,42], significantly affecting the resistive and capacitive elements of the biological tissues, it can be understood that integration with diameter data contributed to correcting variations in bioimpedance caused by different sizes. This suggests that including diameter data when classifying the storage temperature or quality of various-sized mandarins based on BIS can also improve the performance of the ML models. These results align with a previous study that confirmed significant improvement in strawberry ripeness classification by integrating diameter data with bioimpedance for ML models [28].

Additionally, by leveraging the electrical properties of plant tissues, we can represent bioimpedance data with an equivalent circuit (EC) comprising five circuit elements, as depicted in Figure 2. The distributions of the 7 EC parameters are presented in Figure S1. This serves as a dimensionality reduction strategy [24], encapsulating the structural features of plant tissue while condensing the complexity of bioimpedance data (30 impedance and phase values respectively) into 7 circuit parameters. We then trained the ML models with these EC parameters and compared the classification accuracies with those of the bioimpedance-based dataset.

For the EC parameter-based dataset, the classification accuracies were mostly lower than those of the bioimpedance-based dataset. The highest accuracy was 0.82 with the SVM and the MLP model, which was much lower than 0.86 with the bioimpedance data-based SVM or MLP model. This was because there were several constraints in the circuit fitting process. First, it is difficult to accurately model the electrical EC for a certain fruit, which can lead to errors during the fitting process. Second, the fitting result can vary depending on the initial values of the EC parameters. The current method of the EC fitting process involves the interpreter selecting a starting point to kick off, followed by the optimization algorithm finding a minimum point to determine the EC parameters [45]. Thus, a manual adjustment to manage parameter calculation is required for each measurement, making the process dependent on the interpreter. Lastly, adjusting the initial values of various circuit elements can be time-consuming [46]. However, obtaining accurate EC parameters can be utilized as reduced data for the classification model and enable the extension of physiological interpretations based on bioimpedance data. Recent studies in the field of electrochemistry have shown the potential of deep learning in selecting appropriate equivalent circuits and determining their parameters [45,46] and it could be applied to obtain accurate EC parameters for plant tissue’s equivalent circuits to improve the classification model.

**Figure 7:**
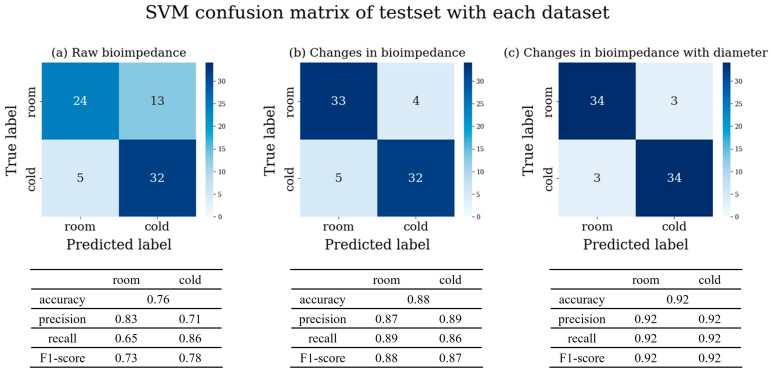
SVM confusion matrix, accuracy, precision, recall, and F1-score of the test set (20% of all experiment data) for storage classification using three types of the dataset (**a**) raw bioimpedance, (**b**) changes in bioimpedance, (**c**) changes in bioimpedance with diameter.

We obtained the confusion matrix for the SVM model trained with the bioimpedance-based dataset, which showed statistically significant performance improvement and the highest accuracy among all models, as determined by a Wilcoxon signed-rank test, and calculated the precision, recall, and F1-score using Equations (6) and (7) for the test set in Figure 7. The same figure for the training set is shown in Figure S3. The model trained with raw bioimpedance data (Figure 7a) showed a high recall value of 0.86 for predicting the cold group. However, the recall value for the room group was 0.65, indicating that the samples stored at room temperature were incorrectly predicted as cold storage samples. Furthermore, the model is difficult to trust, with overall accuracy being relatively low at 0.76.

The SVM model using the changes in bioimpedance (Figure 7b) showed a significant improvement in the room group’s recall compared to the model using raw bioimpedance, reaching 0.89. Additionally, the classification accuracy improved from 0.76 to 0.88.

By integrating both changes in bioimpedance and diameter data (Figure 7c), we achieved a further improvement. This model demonstrated an accuracy of 0.92, the highest among all the datasets. The high accuracy, precision, recall, and F1-score of this model, as indicated in Figure 7c, underscore its reliability. In conclusion, we developed a highly accurate and reliable model for classifying the storage temperature of mandarins.

Many studies have applied BIS to various fruits such as avocados [25], tomatoes [26], strawberries [27,28], bananas [29], grapefruit [30], lemon [31], tangerine [20], and orange [32]. However, only one recent study [28] has considered fruit diameter when applying BIS. Therefore, this study confirms that both the use of changes in bioimpedance and the use of diameter data can individually enhance the performance of BIS-based ML models and that their combined application can further improve the model’s performance. This approach could also be applied to various fruits studied in previous research. Furthermore, including this study, previous research on BIS has rarely examined its correlation with fruit quality indicators such as acidity and total suspended solids. Therefore, future studies could benefit from incorporating these indicators to further explore and expand the potential of BIS in fruit quality assessment.

The freshness of mandarins is greatly affected by storage temperature [6], but changes in freshness are difficult to determine by their appearance. Therefore, in this study, a method for classifying mandarin storage temperature using non-destructive BIS can help determine appropriate pricing based on freshness in distribution and consumption, contributing to rational consumer decisions. In this study, commercial hydrogel electrodes were used and attached to the same location on the mandarin surface to obtain continuous bioimpedance data. However, this method has limitations, as the hydrogel dries out over time, causing signal drift, and making it unsuitable for prolonged attachment. Additionally, ensuring consistent placement is challenging, which may lead to variations in BIS data by attachment location.

Recently, many studies have developed bio-compatible electrodes [47,48,49]. Because bioimpedance data can be affected by contact resistance between electrodes and biological tissues, as well as by non-homogeneous sample variation in biological tissues, these wearable electrodes could promise higher performance in analyzing time-series bioimpedance changes and hold potential for various applications in plants and fruits. Furthermore, by integrating with previous studies that attached strain gauges to the surface of plant bodies to monitor fruit size growth [50], the diameter data could be easily incorporated with bioimpedance to enhance classification accuracy. In future studies, such wearable plant electrodes could be modularized to enable Wi-Fi-based communication using a simple microcontroller, such as a Raspberry Pi, and integrated with cloud platforms to facilitate real-time plant monitoring [51].

## 4. Conclusions

In this study, we obtained bioimpedance data using non-invasive hydrogel electrodes to classify the storage temperature of mandarins. We confirmed that integrating the mandarin’s diameter with changes in bioimpedance is effective for enhancing classification accuracy comparing raw bioimpedance-based datasets. Also, we examined the possibility of EC parameters as inputs for training models. Then, we constructed six datasets and compared the accuracy based on eight ML models to identify the best model and types of datasets. For the models trained with raw bioimpedance data, SVM, LR, and MLP achieved the highest accuracy with 5 times repeated 10-fold CV results of 0.81, 0.77, and 0.79 respectively. When trained with the changes in bioimpedance data, the classification accuracies increased for most ML models except for LDA. The SVM model trained with changes in bioimpedance and diameter achieved the highest cross-validation accuracy of 0.86. The SVM model exhibited the highest accuracy with the test dataset, increasing from 0.76 to 0.92 by incorporating both changes in bioimpedance and diameter data. Although the models trained with EC parameter data showed no considerable improvements in accuracy compared with bioimpedance-based models, there is a potential to improve using deep learning methods in the future [45,46]. These findings highlight the potential of integrating the changes in bioimpedance and the diameter of fruits to enhance the storage temperature classification performance when employing BIS. The classification of mandarin storage temperature based on non-destructive BIS can help determine appropriate pricing based on product value in distribution and consumption settings, contributing to rational consumer decisions.

## Figures and Tables

**Table 1:** Data description for the following six datasets for machine learning models: Bioimpedance and EC parameter-based datasets (raw data, changes, and changes with diameter respectively).

Dataset	Bioimpedance-Based Dataset	EC Parameter-Based Dataset
Raw data	Raw Z and θ at 30 frequency points (368 instances, 60 features)	Seven EC parameters (368 instances, 7 features)
Changes	Changes of Z and θ at 30 frequency points by Equation (4) (368 instances, 60 features)	Changes of seven EC parameters by Equation (4) (368 instances, 7 features)
Changes with diameter	Diameter data column added (368 instances, 61 features)	Diameter data column added (368 instances, 8 features)

**Table 2:** Mean accuracy and standard deviation of 5 times repeated 10-fold cross-validation for eight machine learning models using six types of dataset with determined hyperparameters by grid search method. Different letters indicate significant differences in mean accuracies according to the Wilcoxon signed-rank test (*p* < 0.05). Note that caption letters are independently assigned within each dataset group (bioimpedance and EC parameter).

Dataset		Mean Accuracy (Standard Deviation)							
		SVM	LR	MLP	kNN	RF	LDA	NB	DT
Bioimpedance	Raw data	0.81 ^a^ (0.08)	0.77 ^a^ (0.08)	0.79 ^a^ (0.08)	0.74 ^a^ (0.09)	0.77 ^a^ (0.08)	0.77 ^a^ (0.10)	0.72 ^a^ (0.09)	0.68 ^a^ (0.09)
	Changes in bioimpedance	0.85 ^b^ (0.06)	0.80 ^a^ (0.07)	0.83 ^b^ (0.06)	0.79 ^b^ (0.08)	0.82 ^b^ (0.08)	0.77 ^a^ (0.08)	0.75 ^a^ (0.07)	0.75 ^b^ (0.07)
	Changes in bioimpedance with diameter	0.86 ^c^ (0.06)	0.81 ^a^ (0.07)	0.86 ^b^ (0.06)	0.82 ^c^ (0.07)	0.82 ^b^ (0.08)	0.77 ^a^ (0.08)	0.75 ^a^ (0.07)	0.77 ^b^ (0.07)
EC parameter	Raw data	0.75 ^a^ (0.08)	0.73 ^a^ (0.08)	0.73 ^a^ (0.09)	0.70 ^a^ (0.09)	0.73 ^a^ (0.09)	0.72 ^a^ (0.08)	0.72 ^a^ (0.07)	0.67 ^a^ (0.09)
	Changes in EC parameter	0.79 ^b^ (0.06)	0.79 ^b^ (0.07)	0.78 ^b^ (0.08)	0.74 ^b^ (0.07)	0.78 ^b^ (0.07)	0.78 ^b^ (0.07)	0.65 ^b^ (0.09)	0.75 ^b^ (0.10)
	Change in EC parameter with diameter	0.82 ^c^ (0.08)	0.76 ^c^ (0.07)	0.82 ^c^ (0.08)	0.78 ^c^ (0.06)	0.81 ^c^ (0.07)	0.78 ^b^ (0.07)	0.65 ^b^ (0.09)	0.75 ^b^ (0.09)

## Data Availability

Data will be made available on request.

## Supplementary Materials

The following supporting information can be downloaded at: https://www.mdpi.com/article/10.3390/s25082627/s1, Table S1: The hyperparameter combinations for model tuning based on grid search method for each machine learning model; Table S2: 5 times repeated 10-fold cross-validation accuracies of eight machine learning models for principle hyperparameter combinations; Table S3: The determined hyperparameter of each machine learning model. Parameters not considered in the grid search were set to their default values; Figure S1: Box plot of seven equivalent circuit parameters for room (red) and cold (blue) storage. The central line within each box represents the median value, while the edges of the box correspond to the first and third quartiles, and any points outside this range are considered outliers, marked with yellow circles; Figure S2: Loading values of three PCs for four impedance feature groups ($Z_{low},\theta_{low},Z_{high},\theta_{high}$) from two types of dataset (raw bioimpedance (a), (b), (c) and changes in bioimpedance (d), (e), (f)). The subscript “low” refers to frequencies below 1 kHz, and “high” refers to frequencies above 1 kHz. Error bars represent standard deviations across features within each feature group. Letters (a, b, c, d) indicate statistically distinguishable clusters based on Tukey’s HSD tests (*p* < 0.05); Figure S3: SVM confusion matrix, accuracy, precision, recall, and F1-score of the training set (80% of all experiment data) for storage classification using three types of the dataset (a) raw bioimpedance, (b) changes in bioimpedance, (c) changes in bioimpedance with diameter.
