# Supplementary material for: Classifying Storage Temperature for Mandarin (Citrus reticulata L.) Using Bioimpedance and Diameter Measurements with Machine Learning
Source: Sensors (Basel). 2025 Apr 21;25(8):2627. doi: 10.3390/s25082627 (PMC12031307; doi:10.3390/s25082627)
Supplement: Supplementary file 1 [file sensors-25-02627-s001.zip › sensors-3583693-supplementary.pdf]

Supplementary Data

Table S1. The hyperparameter combinations for model tuning based on grid search method for each machine learning model.

ML Model	Hyperparameter combinations
SVM	'kernel': ['linear', 'rbf', 'poly', 'sigmoid'], 'C': [0.1, 1, 10, 20]
LR	'C': [0.1, 1, 10]
MLP	'hidden_layer_sizes': [(3,), (5,), (10,), (3, 3), (5, 3), (10, 3)], 'activation': ['relu', 'tanh']
kNN	'n_neighbors': [3, 5, 7, 9], 'leaf_size': [5, 10, 20, 30, 40, 50, 100], 'p': [1,2,3]
RF	'n_estimators': [25, 50, 100, 200], 'max_depth': [3, 6, 10, 20, 30], 'min_samples_split': [3,6,9], 'min_samples_leaf': [3,6,9]
LDA	'solver': ['svd','lsqr', 'eigen']
NB	Basic Gaussian Navie Bayes was used
DT	'max_depth': [3, 6, 9], 'min_samples_split': [3, 6, 9, 12], 'min_samples_leaf': [3, 6, 9, 12]

Table S2. 5 times repeated 10-fold cross-validation accuracies of eight machine learning models for principle hyperparameter combinations.

SVM

Hyperparameters		Accuracy
kernel	C	
linear	10	0.820
rbf	10	0.859
poly	10	0.775
sigmoid	10	0.675
rbf	0.1	0.792
rbf	1	0.825
rbf	10	0.859
rbf	20	0.858

LR

Hyperparameters	Accuracy
C	
0.1	0.794
1	0.801
10	0.812

MLP

Hyperparameters		Accuracy
acitvation	Hidden_layer	
relu	(10,)	0.861
tanh	(10,)	0.846
relu	(3,)	0.829
relu	(5,)	0.843
relu	(10,)	0.861
relu	(3, 3)	0.830
relu	(5, 3)	0.846
relu	(10, 3)	0.839

kNN

Hyperparameters			Accuracy
n_neighbors	p	leaf_size	
3	3	5	0.813
5	3	5	0.815
7	3	5	0.801
9	3	5	0.806
5	1	5	0.799
5	2	5	0.802
5	3	5	0.815

RF

Hyperparameters		Accuracy
n_estimators	max_depth	
25	30	0.803
50	30	0.818
100	30	0.812
200	30	0.822
200	3	0.799
200	6	0.820
200	10	0.813
200	20	0.817
200	30	0.822

LDA

Hyperparameters	Accuracy
solver	
svd	0.774
lsqr	0.774
eigen	0.774

* No difference

NB

* Basic Gaussian Navie Bayes was used

DT

Hyperparameters		Accuracy
min_samples_leaf	max_depth	
3	6	0.745
6	6	0.767
9	6	0.767
6	3	0.763
6	6	0.767
6	9	0.759

Table S3. The determined hyperparameter of each machine learning model. Parameters not considered in the grid search were set to their default values.

ML Model	Hyperparameter combinations
SVM	kernel = ‘rbf’, C = ‘10’, class_weight = None
LR	solver = 'lbfgs', penalty = 'l2', C=10, max_iter = 1000, multi_class = 'multinomial', class_weight = None
MLP	solver = 'adam', hidden_layer_sizes = (10,), alpha = 1e-5, activation = 'relu', learning_rate = 'constant', batch_size = 'auto', max_iter = 5000
kNN	n_neighbors = 5, weights = ‘uniform’, algorithm = 'auto', leaf_size = 5, p = 3, metric='minkowski'
RF	n_estimators = 200, criterion = 'gini', max_depth = 30, min_samples_split = 3, min_samples_leaf = 3, max_features = 'sqrt', max_samples = None, bootstrap = True
LDA	solver = 'svd', shrinkage = None, n_components = None
NB	Gaussian naïve bayes model used, var_smoothing = 1e-9
DT	max_depth = 6, criterion = ‘gini’, splitter = ‘best’, min_samples_split = 6, min_samples_leaf = 6, max_leaf_nodes = None,

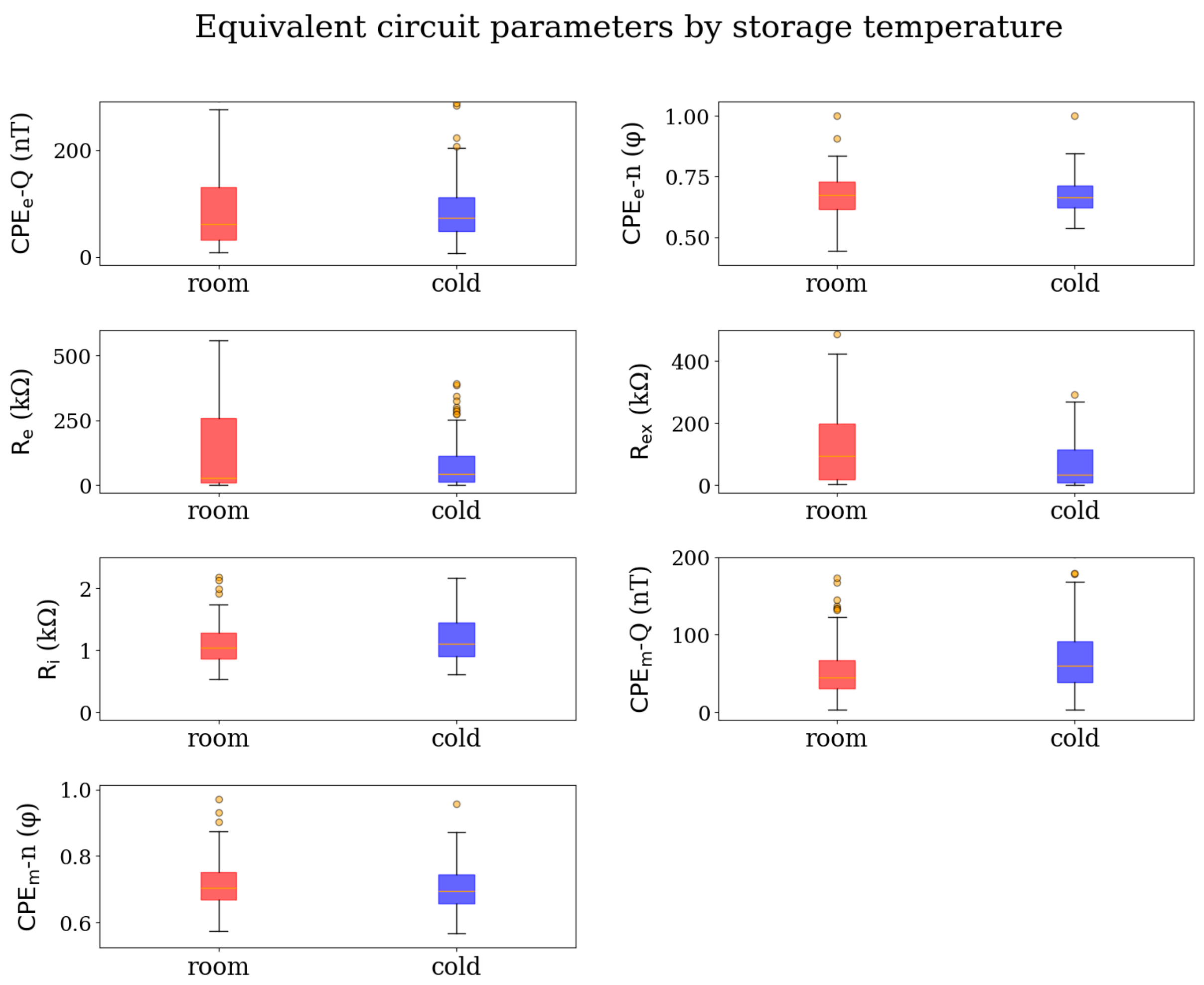

Figure. S1. Box plot of seven equivalent circuit parameters for room (red) and cold (blue) storage. The central line within each box represents the median value, while the edges of the box correspond to the first and third quartiles, and any points outside this range are considered outliers, marked with yellow circles.

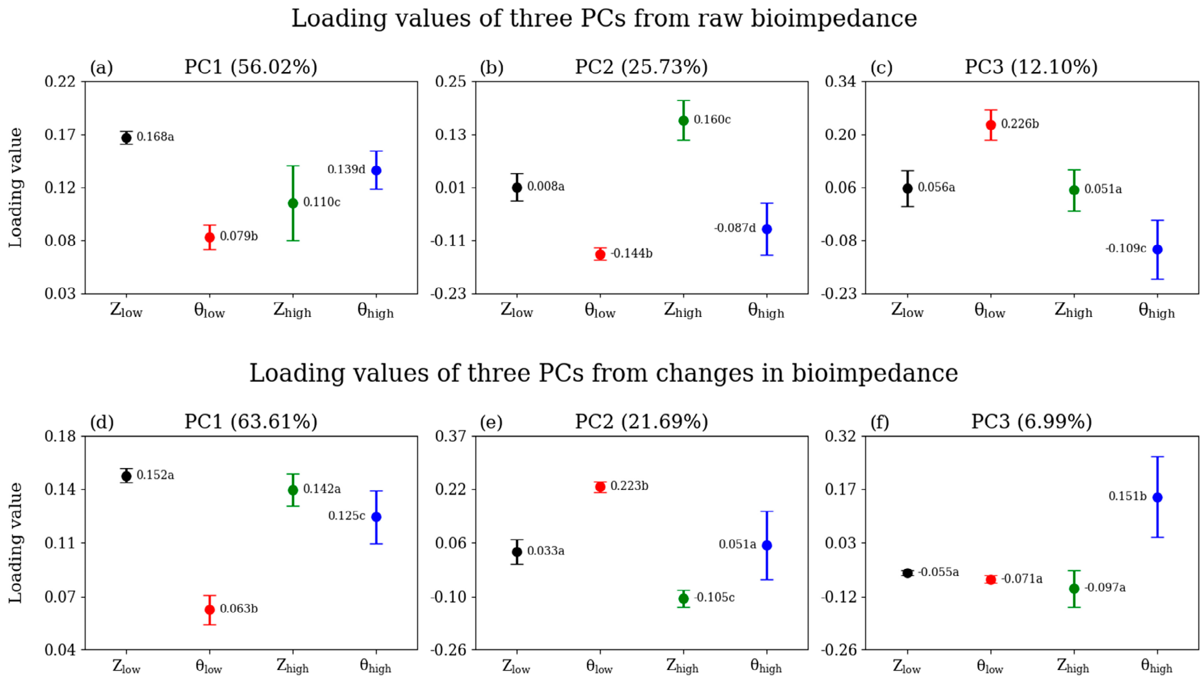

Figure. S2. Loading values of three PCs for four impedance feature groups ($\boldsymbol{Z_{low}}, \boldsymbol{\theta_{low}}, \boldsymbol{Z_{high}}, \boldsymbol{\theta_{high}}$) from two types of dataset (raw bioimpedance (a), (b), (c) and changes in bioimpedance (d), (e), (f)). The subscript "low" refers to frequencies below 1 kHz, and "high" refers to frequencies above 1 kHz. Error bars represent standard deviations across features within each feature group. Letters (a, b, c, d) indicate statistically distinguishable clusters based on Tukey's HSD tests ($p < 0.05$).

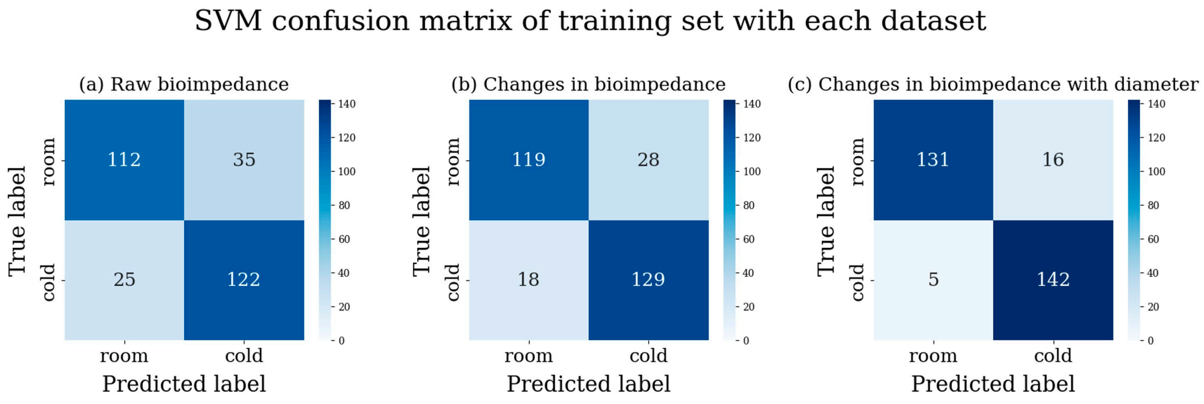

	room	cold
accuracy	0.78	
precision	0.82	0.78
recall	0.76	0.83
F1-score	0.79	0.80

	room	cold
accuracy	0.84	
precision	0.87	0.82
recall	0.81	0.88
F1-score	0.84	0.85

	room	cold
accuracy	0.93	
precision	0.96	0.90
recall	0.89	0.97
F1-score	0.92	0.93

Figure. S3. SVM confusion matrix, accuracy, precision, recall, and F1-score of the training set (80% of all experiment data) for storage classification using three types of the dataset (a) raw bioimpedance, (b) changes in bioimpedance, (c) changes in bioimpedance with diameter.
